# Iniencephaly and Holoprosencephaly: Report of a Rare Association

**DOI:** 10.1155/2014/849589

**Published:** 2014-07-02

**Authors:** Aytekin Tokmak, Hakan Timur, Korkut Dağlar, Özgür Kara

**Affiliations:** Department of Obstetrics and Gynecology, Dr. Zekai Tahir Burak Women's Health Education and Research Hospital, 06240 Ankara, Turkey

## Abstract

The aim of this study is to discuss a rare association of iniencephaly and holoprosencephaly and to state the importance of pregnancy termination in early gestational weeks. An 18-year-old nullipara was admitted to our perinatology service with a diagnosis of neural tube defect. Based on the ultrasonographic findings of alobar holoprosencephaly and iniencephaly during a prenatal screening, termination was recommended at the 13th week of pregnancy. However, she rejected the termination and received no prenatal care until the onset of parturition. At the time of admission, she was in her 28th week of pregnancy. Her medical and family histories were unremarkable. She delivered a stillbirth female weighing 1100 gr complicated with iniencephaly. The infant's postmortem examination showed iniencephaly associated with holoprosencephaly and cyclops. The family declined an autopsy and genetic counseling. In this case, genetics and environmental causes, including lower socioeconomic status and lack of folic acid supplementation, may be risk factors for the current disorder. In conclusion, prenatal diagnosis is possible and termination in early pregnancy should be proposed to patients with iniencephaly associated with holoprosencephaly. In addition, folic acid supplementation should be recommended to reduce the risk of contracting this disorder.

## 1. Introduction

Congenital anomalies are the main causes of perinatal morbidity and mortality. Neurological anomalies and musculoskeletal system malformations are the most frequent disorders found within terminated pregnancies due to congenital anomalies [1]. Iniencephaly is a rare and lethal neural tube defect originally described by Saint-Hilaire in 1836. The classical triad for this disorder is retroflexion of the fetal head, occipital bone defect, and variable rachischisis [2]. The prenatal diagnosis based on ultrasonographic findings may not be adequate for the parents to make the decision to terminate the pregnancy. Therefore, a multidisciplinary approach is required for certain diagnoses of anomalies and for guidance to the parents. Iniencephaly may be associated with other neurological anomalies. Here we present a very rare combination of iniencephaly with holoprosencephaly.

## 2. Case Presentation

**Figure 1:**
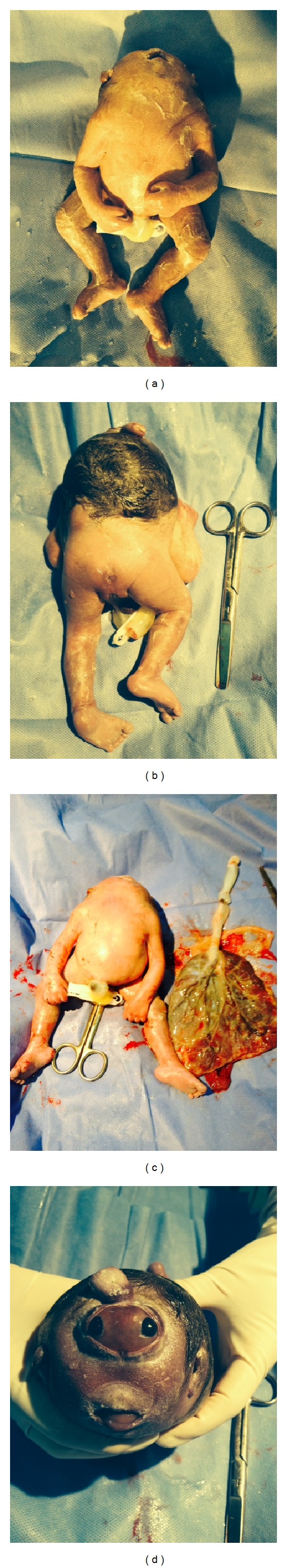
Cervicothoracal lordosis, iniencephaly, and holoprosencephaly.

**Figure 2:**
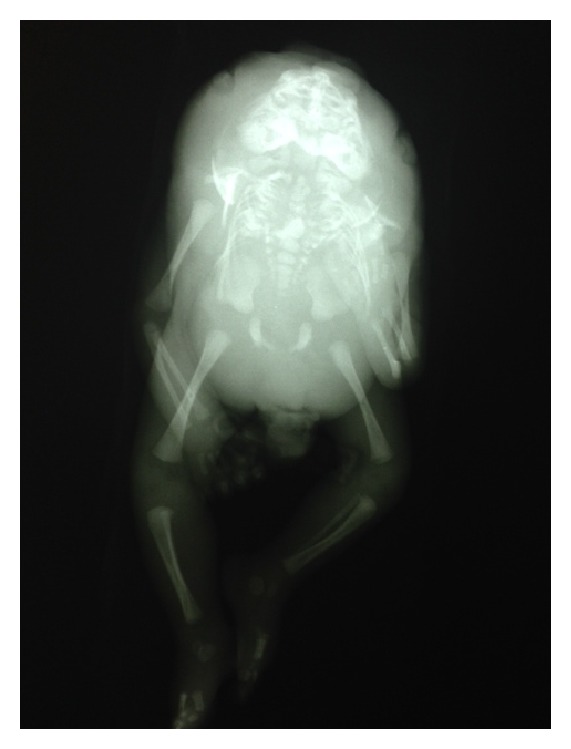
Radiological image of the fetus.

An 18-year-old primipara was admitted to our perinatology service with a diagnosis of breech presentation, preterm birth, and fetal anomaly. She came once to our high-risk pregnancy outpatient clinic in her 13th week of pregnancy. On her first admission, a fetus with a crown-rump length (CRL) measuring 72 mm (at 13 weeks, 2 days gestation) was depicted during a transvaginal sonographic examination, and this was incompatible with her last menstrual period. She had a 2-week delay according to the last menstrual period. Fetal cardiac activity was present. An occipital bone defect and fixed retroflexion of the fetal head were noted. Her detailed neurosonography revealed a single ventricle and fused cerebral hemispheres. Falx cerebri, interhemispheric fissure, and the fissure of Sylvius were absent. There was no pathological sonographic finding in other systems. Based on the findings of alobar holoprosencephaly and iniencephaly during the prenatal ultrasound screening, termination of the pregnancy was recommended. However, she and her family rejected termination due to religious reasons. Afterward, she did not visit another physician until onset of parturition. She was at her 28th gestation week on this admission. Her obstetric and family histories were unremarkable. She was living in low socioeconomic conditions smoking half a pack of cigarettes a day and did not receive any vitamin supplementation. There was no history of substance abuse, drug use, or potential teratogen exposure and also no kinship with her husband. During her vaginal examination, full effacement and dilation were noted and breech presentation was detected. Fetal cardiac activity was ongoing and her contractions were adequate for active labor. She delivered a 1100 gr stillbirth female fetus complicated with iniencephaly. Her postmortem examination showed iniencephaly associated with holoprosencephaly and cyclops (Figure 1). Direct radiography showed a partial absence of the cervicothoracic vertebrae and lordosis (Figure 2). The family declined an autopsy and genetic evaluation.

## 3. Discussion

Iniencephaly is a rare malformation of the central nervous system. It is characterized by fusion of the occiput (inion) and cervical spine, resulting in retroflexion of the head on the spine. Its incidence ranges from 0.1 to 10 in 10,000 [3]. Iniencephaly may be associated with other anomalies such as anencephaly, encephalocele, meningomyelocele, hydrocephalus, Dandy-Walker malformation, holoprosencephaly, omphalocele, congenital diaphragmatic hernia, hydronephrosis, polycystic kidneys, cardiac defects, caudal regression sequence, arthrogryposis, club foot, single umbilical artery, and gastrointestinal atresia [4]. Anencephaly is the most frequently reported anomaly associated with iniencephaly [5]. In a series of cases, iniencephaly associated with holoprosencephaly was reported as 1,6% (one in 63 cases) [6]. The majority of iniencephalic babies are stillborn or die soon after birth; however, milder cases are not lethal. There is a female tendency for iniencephaly [3]. Iniencephaly was generally categorized into iniencephaly apertus and iniencephaly clausus, according to the presence of encephalocele [6]. In our case, which involved a stillborn female fetus, iniencephaly aperta and spina bifida aperta were not noted during the postmortem examination.

The developmental etiopathogenesis of this condition is not well known; both genetic factors and environmental factors may contribute to the condition. Chromosomal abnormalities including trisomy 18, trisomy 13, and Turner syndrome have been associated with this disorder [6]. Phadke and Thakur reported on a case of iniencephaly and alobar holoprosencephaly with trisomy 13 mosaicism [7], which was very similar to our case. Our patient likely also had trisomy 13 mosaicism, but her family rejected the genetic evaluation, so we cannot be sure of this.

Environmental causes such as poor socioeconomic conditions, low parity, lack of folic acid supplementation, diabetes, obesity, and some drugs have been shown to increase the risk of iniencephaly [8]. In this case, lower socioeconomic status and lack of folic acid supplementation during pregnancy may have contributed to the disorder. Another risk factor may be pregnancy in an early or late stage of a woman's reproductive life cycle.

Holoprosencephaly, also known as arhinencephaly and cyclops, is a brain defect resulting from incomplete cleavage of the embryonic forebrain. It is usually presented with facial dismorphism and accompanied by different structural abnormalities. It is characteristically recognized with cyclops, ethmocephaly, cebocephaly, and premaxillary agenesis. However, holoprosencephaly has a broad phenotypic spectrum and manifests in various forms. The prevalence of holoprosencephaly was 1.2 per 10,000 births [9]. The facial phenotype did not strongly predict the severity of the brain defect; however, severity was negatively correlated with length of survival. Most of the affected babies die within a few hours.

Dogan and Ozyuncu reported on a fetus with iniencephaly apertus, alobar holoprosencephaly, and cyclopia diagnosed at the 11th week of gestation [10]. They claimed that this is the earliest diagnosed case of this type, despite the fact that cases in later gestational weeks were reported in the literature. In our case, early prenatal diagnosis of the anomaly was made during an ultrasound scan, but the family rejected termination of the pregnancy. After such a diagnosis in the prenatal period, physicians should provide detailed information to the family about the disorder. The family should then be referred to a geneticist, a psychologist, and a neurologist. When such a pregnancy progresses, the risk of obstetrical complications may increase. Informative materials and photographs such as ours may be supplied to the parents of the fetus to persuade them to terminate the pregnancy.

In this case report, we presented the association between two rare congenital malformations. Iniencephaly is a rare entity and commonly associated with anencephaly, but here it was associated with holoprosencephaly. This condition can cause obstructed labor because of retroflexion of the fetal head in advanced gestational weeks and can recur in subsequent pregnancies [2]. There is a recurrent risk of iniencephaly, especially among families with a history of neural tube defect [11].

## 4. Conclusion

Prenatal diagnosis is possible and termination in early pregnancy should be proposed to patients with iniencephaly associated with holoprosencephaly. Folic acid supplementation should be recommended for reducing a recurrent risk of this disorder.
